# Matrigel induces L-plastin expression and promotes L-plastin-dependent invasion in human cholangiocarcinoma cells

**DOI:** 10.3892/ol.2014.2239

**Published:** 2014-06-11

**Authors:** SUTHIDARAK CHAIJAN, SITTIRUK ROYTRAKUL, APIWAT MUTIRANGURA, KAWIN LEELAWAT

**Affiliations:** 1 Inter-Departmental Program in Biomedical Sciences, Faculty of the Graduate School, Chulalongkorn University, Bangkok 10330, Thailand; 2 The National Center for Genetic Engineering and Biotechnology, National Science and Technology Development Agency, Pathumtani 12120, Thailand; 3 Center for Excellence in the Molecular Genetics of Cancer and Human Diseases, Department of Anatomy, Faculty of Medicine, Chulalongkorn University, Bangkok 10330, Thailand; 4 Department of Surgery, Rajavithi Hospital, Bangkok 10400, Thailand

**Keywords:** cholangiocarcinoma, extracellular matrix, cancer cell invasion, L-plastin, liquid chromatography-tandem mass spectrometry

## Abstract

The function of the extracellular matrix (ECM) in the tumor microenvironment is not limited to forming a barrier against tumor invasion. As demonstrated in pathological specimens, cholangiocarcinoma samples exhibit an enrichment of the ECM surrounding the tumor cells. In this study, we examined involvement of the ECM in the regulation of the invasiveness of cholangiocarcinoma cells. The RMCCA1 cholangiocarcinoma cell line was cultured in culture plates either with or without a coating of reconstituted ECM basement membrane preparation (BD Matrigel matrix). *In vitro* invasion assays were then performed. In addition, the protein expression profile of the cell line was examined using two-dimensional gel electrophoresis and liquid chromatography-tandem mass spectrometry. The proteins expressed and their functional associations with cancer progression were determined. Culturing the RMCCA1 cell line in the BD Matrigel matrix induced cell invasion. Numerous proteins were induced by culturing the RMCCA1 cells in the matrix gel. The expression of L-plastin, an actin-binding protein, was significantly upregulated. The knockdown of L-plastin expression by siRNA silencing significantly suppressed the cellular response to matrix gel-stimulated cancer cell invasion. The ECM promotes the invasiveness of cholangiocarcinoma cells by upregulating L-plastin. These findings suggest the potential exploitation of this mechanism as a means of inhibiting the invasiveness of cholangiocarcinoma cells.

## Introduction

Cholangiocarcinoma, an aggressive malignant tumor that develops from the bile duct epithelium, is associated with local invasiveness and a high rate of metastasis (1,2). The worldwide incidence and mortality rates associated with cholangiocarcinoma have risen over the past three decades. In Thailand, the annual incidence of cholangiocarcinoma is 87 per 100,000 inhabitants (3). In the United States, the most commonly recognized risk factor for cholangiocarcinoma is primary sclerosing cholangitis (4). However, in Southeast Asia and particularly in Thailand, infection with hepatobiliary flukes (*Opisthorchis viverrini*) is the most common risk factor for cholangiocarcinoma (5). Therapeutic options for cholangiocarcinoma patients are limited, as this type of cancer responds poorly to chemotherapy and radiation therapy. Surgery is thus the only potentially effective treatment for cholangiocarcinoma. However, typical five-year survival rates of 32–50% are achieved only by a small number of patients with negative histological margins at the time of surgery (6–8). Therefore, the understanding of the mechanisms involved in cancer cell invasion and metastasis may be useful in developing new therapeutic options for cholangiocarcinoma patients.

The function of the extracellular matrix (ECM) in the tumor microenvironment is not limited to forming a barrier against tumor invasion. Previous studies have indicated that interactions between cancer cells and the ECM play an important role in cancer progression. The molecular components of the ECM, such as fibronectin, laminin, collagen and heparin sulfate proteoglycans, communicate with cancer cells and modulate a variety of cellular functions required for cancer cells to exhibit invasive and metastatic properties (9–11). Numerous results from pathological studies have indicated that cholangiocarcinoma cells are surrounded by a dense sheath of connective tissue that contains the ECM (12–14). However, there have been no studies to date regarding the definitive role that the ECM plays in cholangiocarcinoma cell invasion. Therefore, we aimed to investigate the involvement of the ECM in cholangiocarcinoma cell invasion.

## Materials and methods

### Cell cultures

The RMCCA1 human cholangiocarcinoma cell line, originally derived from a cholangiocarcinoma patient (15), was grown in Ham’s F12 medium (Gibco, Grand Island, NY, USA) supplemented with 10% fetal bovine serum (Gibco) at 37°C in a 5% CO_2_ humidified atmosphere.

### Cell invasion assay

To study the mechanism of cancer cell invasion *in vitro*, RMCCA1 cells were cultured in BD Matrigel matrix (BD Biosciences, Bedford, MA, USA) for 0–24 h. Next, cancer cells were seeded into porous cell culture insert cups (BD Biosciences) each containing a layer of matrix gel. The number of cancer cells that invaded through the basement membrane within 24 h was assessed by staining the cells with crystal violet (Sigma-Aldric, St. Louis, MO, USA) (16).

### Two-dimensional (2D) gel electrophoresis

2D gel electrophoresis was performed for the analysis of proteins extracted from cholangiocarcinoma cells cultured in uncoated and 24-h matrix gel-coated plates. Each electrophoresis gel contained three pooled samples from the cell culture plates. Six gels were prepared in biological triplicates from the uncoated and matrix gel-coated plates. Protein samples (500 μg) were applied to 18-cm immobilized pH gradient (IPG) gel strips (pH 3–10; GE Healthcare, Uppsala, Sweden) by cup loading near the anodic ends of the strips. Isoelectric focusing (IEF) was performed using an Ettan IPGphor Manifold on an Ettan IPGphor isoelectric focusing unit (GE Healthcare) for 32,000 Vh at 20°C. Following IEF, each gel strip was equilibrated with equilibration buffer. The IPG strips were then loaded and run on 12.5% acrylamide gels (GE Healthcare) using the Ettan DALTsix electrophoresis system (GE Healthcare). The run was stopped after the bromophenol blue dye front had run off the bottom of the gels. The gels were then stained with colloidal Coomassie Blue (GE Healthcare).

### 2D image analysis

The proteins were visualized using an ImageScanner (GE Healthcare). The gel images were analyzed to determine differential protein expression profiles using ImageMaster 2D Platinum software (GE Healthcare). Student’s t-test was used for statistical analysis and P<0.05 was considered to indicate a statistically significant difference.

### Protein identification by liquid chromatography-tandem mass spectrometry (LC-MS/MS)

#### In-gel digestion

LC-MS/MS was performed by the Proteomics Laboratory, Genome Institute, National Science and Technology Development Agency (Pathumthani, Thailand). Following 2D analysis, an in-gel digestion was performed. Briefly, after the protein spots were excised, the gel plugs were dehydrated with 100% acetonitrile (ACN), reduced with 10 mM DTT in 10 mM ammonium bicarbonate at room temperature for 1 h and alkylated at room temperature for 1 h in the dark in the presence of 100 mM iodoacetamide in 10 mM ammonium bicarbonate. Following alkylation, the gel pieces were dehydrated twice with 100% ACN for 5 min. For the in-gel digestion of the proteins, 10 μl trypsin solution (20 ng/μl trypsin in 50% ACN/10 mM ammonium bicarbonate) was added to the gels followed by incubation at room temperature for 20 min. Next, 20 μl 30% ACN was added to keep the gels immersed throughout digestion. The gels were incubated at 37°C overnight. To extract the peptide digestion products, 30 μl 50% ACN in 0.1% formic acid was added to the gels, which were then incubated at room temperature for 10 min in a shaker. The extracted peptides were collected and pooled in a new tube. The pooled extracted peptides were dried by vacuum centrifugation at 2,500 × g for 10 min and stored at −80°C until further mass spectrometric analysis.

#### LC-MS/MS analysis

The LC-MS/MS analysis of the digested peptide mixtures was performed using a Waters SYNAPT™ HDMS™ system (Waters, Milford, MA, USA). The 1D-nanoLC was performed with a Waters nanoACQUITY UPLC system (Waters). Tryptic digests (4 μl) were injected onto an reversed-phase analytical column (20 cm × 75 μm) packed with 1.7-μm ethylene bridged hybrid C18 material (Waters). The peptides were eluted with a linear gradient of 2–40% acetonitrile developed over 30 min at a flow rate of 1000 nl/min. This elution was followed by a 10-min 80% acetonitrile treatment to clean the column before using 2% acetonitrile for the next sample. The effluent samples were electrosprayed into a mass spectrometer (SYNAPT HDMS system) for MS/MS analysis of the peptides, and spectral data were generated for further protein identification by matching against hits in a database search.

Mass lists in the form of Mascot generic files were created and used as the inputs for the Mascot MS/MS Ion web-based search functionality at the National Center for Biotechnology Information non-redundant database (www.matrixscience.com). The default search parameters were applied as follows: Enzyme, trypsin; taxonomy, *Homo sapiens* (human); maximum missed cleavages, 1; fixed modifications, carbamidomethyl (C); variable modifications, oxidation (M); peptide tolerance, ±1.2 Da; MS/MS tolerance, ±0.6 Da; peptide charge, 1+, 2+ and 3+; and instrument, ESI-QUAD-TOF.

#### Western blot analysis

Protein extracts isolated from the cells cultured in the uncoated and 24-h matrix gel-coated plates were separated by 12% SDS-PAGE and then transferred onto a nitrocellulose membrane (GE Healthcare). The membrane was subsequently incubated with monoclonal antibodies against L-plastin (1:50; Santa Cruz Biotechnology, Santa Cruz, CA, USA) and β-actin (1:500; Cell Signaling Technology, Danvers, MA, USA). Horseradish peroxidase-conjugated anti-mouse immunoglobulin G (IgG) and anti-rabbit IgG at 1:5,000 dilutions were used as secondary antibodies (GE Healthcare). The blots were visualized using an ECL Plus detection kit and Hyperfilm ECL (GE Healthcare). The western blot results were quantified using densitometer and image analysis software (ImageScanner III and ImageQuant TL; GE Healthcare, Uppsala, Sweden).

#### Inhibition of L-plastin expression using transient siRNA transfection

L-plastin siRNA (Santa Cruz Biotechnology) was used to knock down L-plastin gene expression. A fluorescein-labeled, double-stranded RNA duplex (BLOCK-iT™ Fluorescent Oligo; Invitrogen, Melville, NY, USA) was designed as a control. The siRNA molecules were diluted in Opti-MEM^®^ I Medium without serum (Gibco) and mixed gently. Next, Lipofectamine™ 2000 (Invitrogen) was diluted in Opti-MEM I Medium without serum, mixed gently and incubated for 5 min at room temperature. The diluted siRNA molecules and diluted Lipofectamine 2000 were then combined. The mixtures were incubated for 20 min at room temperature to allow for complex formation to occur. The siRNA molecule-Lipofectamine 2000 complexes were added to each well containing cells and medium, and mixed gently by rocking the plate back and forth. The cells were incubated at 37°C in a CO_2_ incubator for 6 h. Next, the growth medium was replaced after 6 h, and the cells were harvested 24 h after transfection. Western blotting analysis using the L-plastin antibody was performed to assess the degree of L-plastin gene expression knockdown.

#### Immunofluorescence microscopy

Cells (5×10^4^) were incubated with the primary antibody, anti-L-Plastin (1:10), for 1 h at room temperature. The cells were then washed and incubated with the appropriate secondary antibody (Alexa Fluor 594, anti-mouse; Molecular Probes, Grand Island, NY, USA) for 1 h at room temperature. The actin filaments (F-actin) in the cell cytoplasm were stained with Alexa Fluor 488 phalloidin (Molecular Probes), and the nuclei were stained with TOPO3 (Molecular Probes). The cover slides were removed from the plates and mounted with antifade on the slides. Cell images were captured with a confocal scanning biological microscope (FV1000; Olympus, Tokyo, Japan).

#### Immunohistochemical staining

The study was performed with approval from the Ethics Committee of Rajavithi Hospital (Bangkok, Thailand). Paraffinized sections on glass slides were subjected to L-plastin detection by standard immunohistochemical technique. Sections were hybridized overnight at 4°C with a 1:50 dilution of L-plastin antibody (Abcam, Cambridge, MA, USA), followed by incubation with the secondary antibody, polyclonal Goat Anti-Mouse IgG (Abcam), conjugated to horseradish peroxidase that catalyzes a color-producing reaction (Abcam). The signals were visualized under high power magnification (x200) on an Olympus BH2 microscope.

#### Statistical analysis

Continuous values for the observed levels in the invasion assay were expressed as the mean and standard deviation. One-way analysis of variance was used for the analysis of the multiple variables of the cell invasion assay. Student’s t-test was employed to evaluate the mean differences in the intensity volume of each corresponding spot between the two groups of samples. The statistical analysis of the immunohistochemical studies was performed using either the χ^2^ test or Fisher’s exact test. P<0.05 was considered to indicate a statistically significant difference.

## Results

### Culturing cholangiocarcinoma cells in matrix gel increases their invasiveness

**Figure 1:**
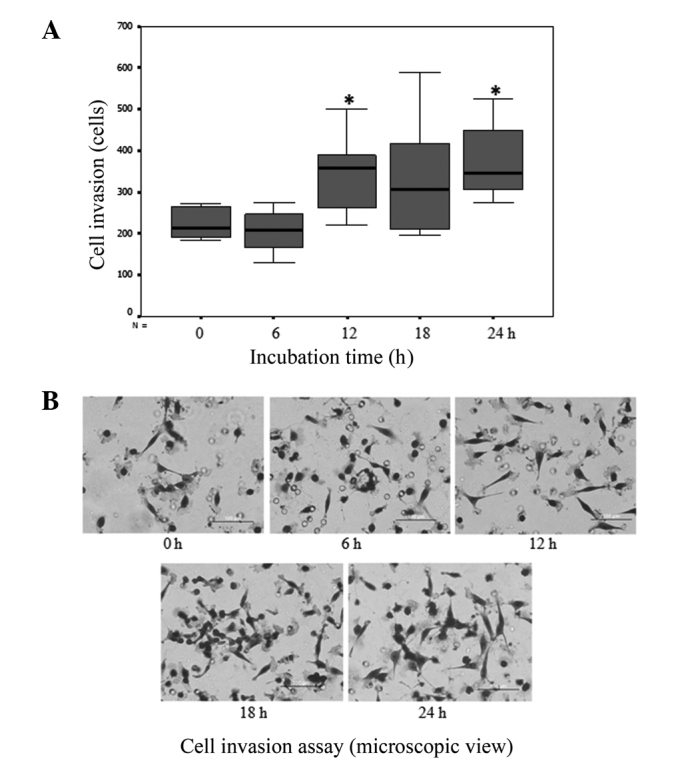
RMCCA1 cholangiocarcinoma cell invasion assays. (A) Box plots comparing the number of cholangiocarcinoma cell invasion events in cells cultured in matrix gel and controls (^*^P<0.001 by analysis of variance, compared with the control). (B) Micrographs of cholangiocarcinoma cell invasion (cholangiocarcinoma cells were cultured in matrix gel for 0, 6, 12, 18 or 24 h before the invasion assays were performed). The scale bar indicates 100 μm (magnification, ×10).

RMCCA1 cholangiocarcinoma cells were incubated in matrix gel for 0–24 h, and invasion assays were then performed. The results showed that a significantly higher number of cholangiocarcinoma cells that were cultured in matrix gel invaded through the insertion cup compared with that observed with the cells that were cultured on uncoated plates (P<0.001; Fig. 1).

### Proteomic study of cholangiocarcinoma cells cultured in matrix gel

To investigate the proteins potentially involved in cholangiocarcinoma cell invasion, cholangiocarcinoma cells were cultured in plates coated with or without matrix gel. Next, 2D gel electrophoresis using pH 3–10 Linear IPG strips was performed to identify the protein expression profiles of these cells. Approximately 800 protein spots were detected by colloidal Coomassie staining. Quantitative intensity and statistical analyses identified 129 protein spots with significantly altered expression levels in matrix gel culture compared with the uncoated culture system. Of these 129 proteins, 60 proteins exhibited greater than two-fold upregulation as determined by mass spectrometry. All the identified proteins were in the expected ranges of their theoretical molecular masses and pI values (Table I). We report for the first time that the ECM plays a major role in the regulation of cholangiocarcinoma cell invasion. Based on 2D electrophoresis results, we identified the proteins that were upregulated when cholangiocarcinoma cells were cultured in matrix gel for 24 h.

### Functional studies of protein expression in cholangiocarcinoma cells cultured in matrix gel

**Figure 2:**
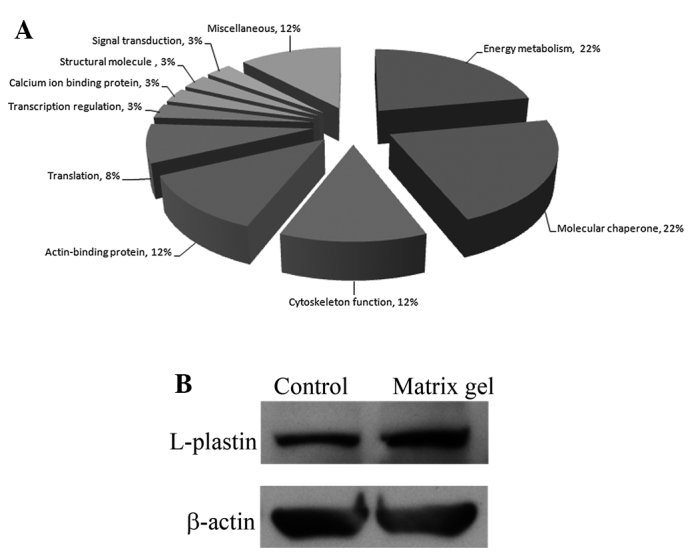
Protein functional analysis in cholangiocarcinoma cells cultured in matrix gel. (A) Functional classification of the differentially expressed proteins in cholangiocarcinoma cells cultured with matrix gel. The altered levels of protein expression were identified by mass spectrometric analysis (see Table I) and categorized according to protein function. Note that the spots with the same identities were counted as only one spot, and each number represents the percentage among the total number of proteins identified. (B) The expression levels of L-plastin in RMCCA1 cells following culture in matrix gel for 24 h were determined by western blot analysis. β-actin was used as a loading control.

The identified proteins that exhibited significant changes in expression levels were classified using a UniProtKB search for protein functional analysis in the species *Homo sapiens* (human). Based on the search results, these proteins are involved in energy metabolism, molecular chaperoning, cytoskeleton functions, actin binding, translation, transcription regulation, calcium ion binding, cell structure and signal transduction (Fig. 2A). Contact with the ECM and the remodeling of the actin cytoskeleton can drive cancer cell motility and promote invasion. L-plastin is one of the actin-binding proteins that exhibited a high level of protein expression in cholangiocarcinoma cells cultured in matrix gel. We performed a western blot analysis to confirm the results of the proteomic study. The results showed that a high level of L-plastin expression was identified in RMCCA1 cells cultured in matrix gel (Fig. 2B). A previous study demonstrated that L-plastin localizes to actin-rich membrane structures involved in locomotion, adhesion and immune defense, thereby implying that L-plastin is involved in the organization of the actin cytoskeleton (17). In addition, L-plastin has also been detected in solid tumors of epithelial and mesenchymal origin and has been suggested to be involved in cancer cell invasion (18). In line with these observations, we found that the number of cholangiocarcinoma cell invasion events significantly decreased when the expression of L-plastin was inhibited with L-plastin siRNA.

### Effect of L-plastin on cholangiocarcinoma cell invasion

**Figure 3:**
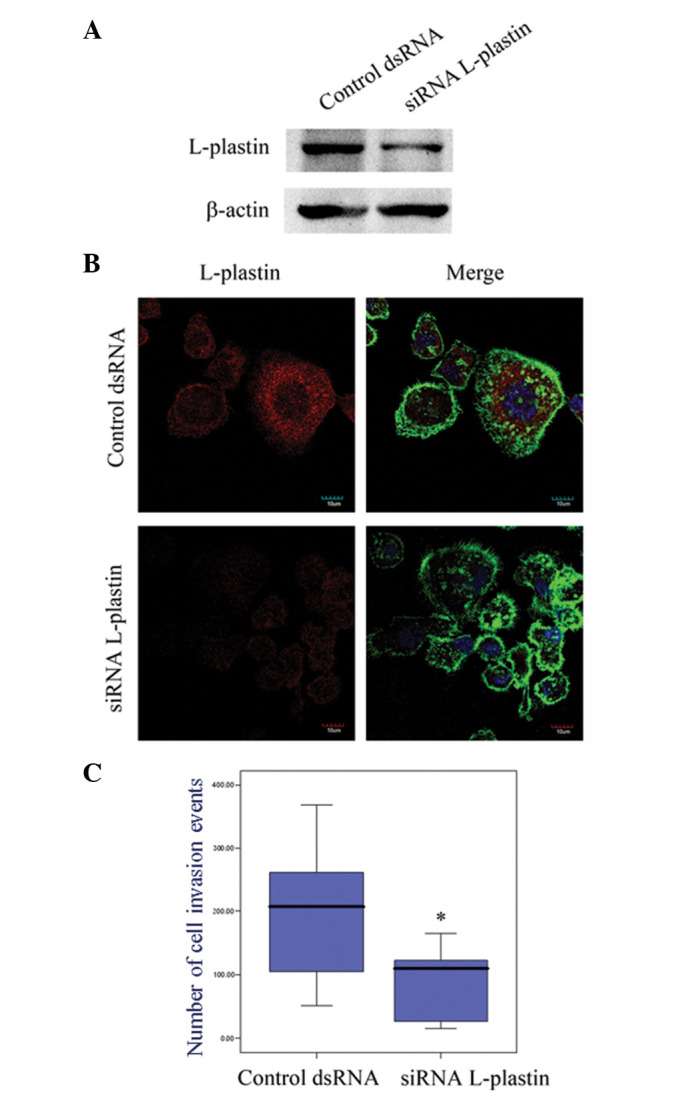
Effect of L-plastin on cholangiocarcinoma cell invasion. (A) The expression levels of L-plastin and β-actin in RMCCA1 cells transfected with either control dsRNA or L-plastin siRNA were determined by western blotting. Lane 1 represents protein extracted from RMCCA1 cells treated with control dsRNA, and lane 2 represents protein extracted from RMCCA1 cells treated with L-plastin siRNA. (B) Immunofluorescence detection by confocal microscopy. The cells were transfected with either control dsRNA or L-plastin siRNA. The cells were then triple-stained with monoclonal L-plastin antibody (red), phalloidin (green, to reveal filamentous actin) or TOPO3 (blue, to reveal the nucleus). The scale bar indicates 10 μm. (C) Box plots comparing the number of cholangiocarcinoma cell invasion events in cholangiocarcinoma cells treated with the control (dsRNA) and L-plastin siRNA (^*^P<0.001 by analysis of variance, compared with the control).

To determine whether the expression of L-plastin is associated with cholangiocarcinoma cell invasion, we knocked down the expression of L-plastin using L-plastin siRNA. The western blot (Fig. 3A) and immunofluorescence studies (Fig. 3B) demonstrated that L-plastin was significantly downregulated after transfecting the RMCCA1 cells with L-plastin siRNA. Moreover, the invasion assay showed that the number of cancer cell invasion events was significantly decreased with the L-plastin siRNA cells compared with those treated with the control dsRNA (P<0.001; Fig. 3C).

### Detection of L-plastin expression in paraffin-embedded cholangiocarcinoma specimens

**Figure 4:**
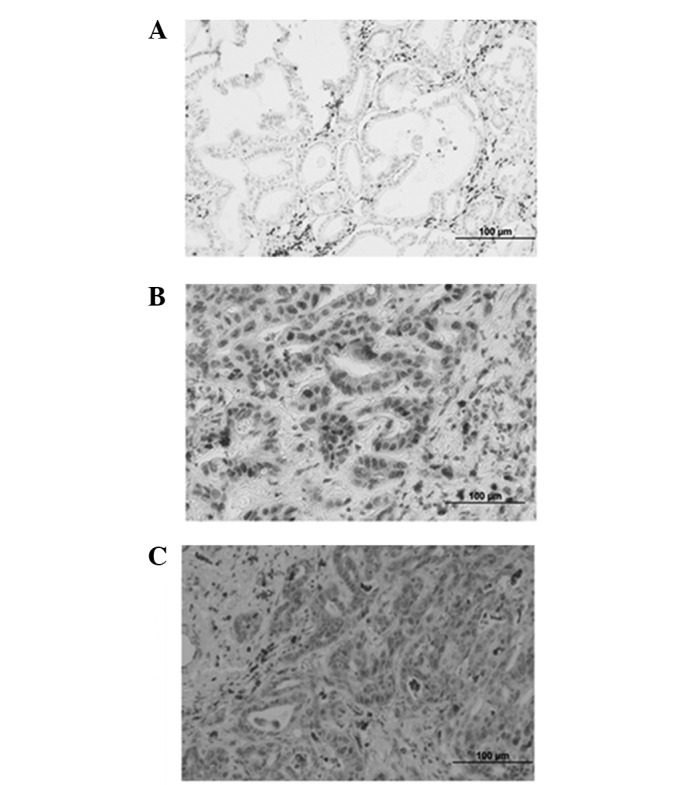
Expression of L-plastin was shown in cholangiocarcinoma specimens by immunohistochemical staining. L-plastin was mainly localized within the nucleus. (A) Negative L-plastin staining; (B) positive L-plastin staining in the nuclei of tumor cells; and (C) positive L-plastin staining in the cytoplasm of tumor cells. The scale bar indicates 100 μm (magnification, ×200).

The expression of L-plastin was determined by immunohistochemistry in 24 paraffin-embedded cholangiocarcinoma specimens. In these cancerous tissues, L-plastin-specific signals were localized mainly in the nucleus and cytoplasm of cholangiocarcinoma cells that invaded the basement membrane and presented as mesenchymal-like cells (Fig. 4). However, cholangiocarcinoma cells that were arranged in a granular structure were negative for L-plastin.

We found that 37.5% (9/24) of the cholangiocarcinoma specimens were positive for the L-plastin expression signal. The correlation between the expression of L-plastin and the clinical characteristics is shown in Table II. The expression of L-plastin in cholangiocarcinoma was detected in all stages of the disease.

## Discussion

We report for the first time that the ECM plays a major role in the regulation of cholangiocarcinoma cell invasion. Based on 2D electrophoresis results, we identified the proteins that were upregulated when cholangiocarcinoma cells were cultured in matrix gel for 24 h. L-plastin, a major F-actin-bundling protein, was significantly upregulated in matrix-gel-coated plates compared with uncoated plates. The results were confirmed by western blotting, as L-plastin exhibited higher expression levels in RMCCA1 cells cultured in matrix-coated plates. A previous study demonstrated that L-plastin localizes to actin-rich membrane structures involved in locomotion, adhesion and immune defense, thereby implying that L-plastin is involved in the organization of the actin cytoskeleton (17). In addition, L-plastin has also been detected in solid tumors of epithelial and mesenchymal origin and has been suggested to play a role in cancer cell invasion (18). In line with these observations, we found that the number of cholangiocarcinoma cell invasion events significantly decreased when the expression of L-plastin was inhibited with L-plastin siRNA.

Confirming the results of prior studies, we observed that L-plastin is located in the nuclei and cytoplasm of cancer cells (17,19). The functional relevance of the nucleocytoplasmic shuttling of L-plastin currently remains unclear. L-plastin may be involved in the regulation of nuclear actin, which is an essential component of the pre-initiation complex and cooperates with polymerases I, II and III in the regulation of gene expression (20). The formation of protrusive structures is driven by spatially and temporally regulated actin polymerization at the leading edge of the cell (21). Further studies should be performed to elucidate the involvement of L-plastin localization in cholangiocarcinoma cells. We found that L-plastin was primarily expressed in mesenchymal-like cholangiocarcinoma cells. These findings suggest that L-plastin expression is associated with the epithelial-mesenchymal transition of cholangiocarcinoma cells.

To understand whether our *in vitro* findings are also relevant *in vivo*, we performed immunohistochemical analyses of tumor specimens derived from cholangiocarcinoma patients. Our analyses demonstrated that L-plastin is expressed in cholangiocarcinoma specimens. However, the level of expression was not significantly correlated with tumor differentiation, lymph node or metastatic status. This finding is in contrast to that reported for colorectal cancer, in which the expression of L-plastin is significantly correlated with cancer staging (22). Variations in the biological features of the tumors and the limited number of specimens in our study may account for these differences. In conclusion, attachment to the ECM promotes cholangiocarcinoma cell progression by inducing L-plastin expression. Understanding this mechanism may help to identify a novel molecular target for the development of an effective therapy for cholangiocarcinoma patients.

## Figures and Tables

**Table I:** A summary of upregulated proteins expressed in cholangiocarcinoma cells cultured in matrix gel, as identified by Q-TOF MS and MS/MS analyses.

Functional category and protein name	GI number	Mr	pI	Score	Coverage %	Ratio	Gene ID	Cellular component
Actin-binding protein								
L-plastin	62087548	56,196	5.21	52	6	4.6	LCP1, PLS2	Cytoplasm, cell membrane, cytoskeleton
Cytovillin 2 (Ezrin)	340217	68,233	5.80	335	13	3.9	VIL2, EZR	Cytoplasm, cell membrane, cytoskeleton
ARP3 actin-related protein 3 homolog	5031573	47,797	5.61	150	6	3.5	ACTR3	Cytoskeleton
α-actinin-4	2804273	102,661	5.27	72	2	3.0	ACTN4	Cytoplasm, nucleus
Adenylyl cyclase-1 associated protein	116241280	52,222	8.27	54	10	2.6	CAP1	Cell membrane
Fascin	4507115	55,123	6.84	160	19	2.5	FSCN1	Cytoplasm, cytoskeleton
Cofilin-1	5031635	18,719	8.22	129	17	2.3	CFL1	Cytoplasm, nucleus, cell membrane, cytoskeleton
Energy metabolism								
Pyruvate Kinase (Pkm2)	67464392	60,277	8.22	166	20	7.3	PKM2	Cytoplasm, nucleus
Phosphoglycerate kinase 1	4505763	44,985	8.30	439	20	6.3	PGK1, PGKA	Cytoplasm
Aldolase A	28614	39,706	8.34	267	11	3.5	ALDOA, ALDA	Cytoplasm, nucleus
α-enolase (phosphopyruvatehydratase)	693933	47,421	7.01	148	22	2.4	ENO1	Cytoplasm, nucleus
L-lactate dehydrogenase A chain	126047	36,950	8.44	85	18	2.3	LDHA, PIG19	Cytoplasm
ATP synthase, H^+^ transporting, mitochondrial F1 complex	4757810	59,828	9.16	182	17	13.5	ATP5A1, ATP5F1	Mitochondrion, Mitochondrion inner membrane
Dihydrolipoamidesuccinyl transferase	643589	48,896	8.90	165	7	3.8	DLST	Mitochondrion
Citrate synthase	33337556	51,942	8.45	169	5	3.4	CS	Mitochondrion
Fumaratehydratase, mitochondrial	182794	50,524	7.23	305	11	2.7	FH	Mitochondrion, cytoplasm
Glutamate dehydrogenase	4885281	61,701	7.66	197	16	3.9	GLUD1	Mitochondrion
Dihydrolipoamide dehydrogenase	83753870	50,656	6.50	236	9	2.2	DLD	Mitochondrion
acyl-Coenzyme A dehydrogenase	76496475	68,414	8.76	233	10	2.1	ACADVL	Mitochondrion, mitochondrion inner membrane
Transketolase	37267	68,435	7.90	102	16	2.8	TKT	Cytoplasm, nucleus
Molecular chaperone								
Tumor rejection antigen 1, Endoplasmin	74755280	92,282	4.77	89	4	11.9	GRP94, TRA1, HSP90B1	Endoplasmic reticulum
T-complex protein 1 subunit γ	14124984	60,934	6.10	157	7	6.3	CCT3, CCTG	Cytoplasm
Heat shock protein HSP 90-α	154146191	85,006	4.94	163	5	5.2	HSP90AA1	Cytoplasm
Heat shock protein HSP 90-β	119602173	57,868	4.92	59	2	2.3	HSP90AB1	Cytoplasm
Stress-induced-phosphoprotein) 1 (Hsp70/Hsp90-organizing protein	5803181	63,227	6.40	145	19	3.8	STIP1	Cytoplasm, nucleus
60 kDa heat shock protein	77702086	61,346	5.70	580	16	5.0	HSPD1, HSP60	Mitochondrion
Heat shock protein	386785	70,110	5.42	404	10	4.4	HSPA1L	Cytoplasm
Heat shock 70 kDa protein 8	5729877	71,082	5.37	203	13	3.8	HSPA8	Cytoplasm
Stress-70 protein, mitochondrial	21264428	73,920	5.87	70	6	3.7	HSPA9	Mitochondrion
78 kDa glucose-regulated protein	386758	72,185	5.03	251	7	4.1	GRP78, HSPA5	Endoplasmic reticulum
T-complex polypeptide 1	36796	60,869	6.03	197	9	4.0	TCP1	Cytoplasm, cytoskeleton
Nucleophosmin (nucleolarphosphoprotein B23, numatrin)	15214852	32,760	4.64	198	12	3.3	NPM1, NPM	Cytoplasm, nucleus, cytoskeleton
Calreticulin	4757900	48,283	4.29	109	12	2.3	CALR	Cytoplasm, endoplasmic reticulum, extracellular matrix, secreted
Structural molecule								
Tubulin, β, 2	5174735	50,255	4.79	282	14	3.8	TUBB2C, TUBB4B	Cytoplasm, cytoskeleton, microtubule
α-tubulin	37492	50,810	5.02	123	13	2.6	TUBA4A, TUBA1	Cytoplasm, cytoskeleton, microtubule
Cytoskeleton function								
Keratin, type I cytoskeletal 17	4557701	48,361	4.97	303	28	3.7	KRT17	Cytoplasm, intermediate filament, keratin
Keratin, type I cytoskeletal 18	30311	47,305	5.27	558	28	3.5	KRT18, PIG46 CYK18	Cytoplasm, intermediate filament, keratin
Keratin, type I cytoskeletal 19	24234699	44,079	5.04	455	44	2.1	KRT19	Intermediate filament, keratin
Keratin, type II cytoskeletal 6A	5031839	60,293	8.09	248	29	5.8	KRT6A	Intermediate filament, keratin
Keratin, type II cytoskeletal 2 epidermal	908801	60,448	8.09	422	15	4.3	KRT2	Intermediate filament, keratin
Keratin, type II cytoskeletal 8	181573	53,529	5.52	238	9	4.3	KRT8, CYK8	Cytoplasm, Intermediate filament, keratin, nucleus
Keratin, type II cytoskeletal 7	12803727	51,444	5.42	287	36	3.5	KRT7	Cytoplasm, intermediate filament, keratin
Transcription regulation								
Far upstream element-binding protein 1	17402900	67,690	7.18	111	4	4.1	FUBP1	Nucleus
ETS translocation variant 5	221042722	65,643	5.69	174	9	2.6	ERM	Nucleus
Translation								
Ribosomal protein P0	4506667	34,423	5.71	193	14	3.5	RPLP0	Cytoplasm, nucleus
Tyrosyl-tRNAsynthetase	4507947	59,448	6.61	403	16	2.4	YARS	Cytoplasm
Heterogeneous nuclear ribonucleoprotein L	11527777	64,617	8.49	173	6	3.9	HNRNPL, HNRPL, P/OKcl.14	Cytoplasm, nucleus
Heterogeneous nuclear ribonucleoproteins A2/B1	4504447	36,041	8.67	188	13	3.2	HNRNPA2B1	Cytoplasm, nucleus, spliceosome
Heterogeneous nuclear ribonucleoprotein K	460789	51,325	5.13	88	12	2.0	HNRNPK, HNRPK	Cytoplasm, nucleus, spliceosome
Calcium ion binding protein								
Annexin A1	4502101	38,918	6.57	124	26	3.9	ANXA1, ANX1, LPC1	Cytoplasm, nucleus, cell membrane
Annexin A2	56967118	36,634	8.32	210	13	2.1	ANXA2	Basement membrane, extracellular matrix
Signal transduction								
14-3-3 protein epsilon	5803225	29,326	4.63	136	26	2.5	YWHAE	Cytoplasm
14-3-3 protein β/α	4507949	28,179	4.76	315	23	2.2	YWHAB	Cytoplasm
Elongation factor								
Elongation factor Tu	704416	49,851	7.70	229	19	3.9	TUFM	Mitochondrion
Proteasome regulatory								
26S proteasome non-ATPase regulatory subunit 12	4506221	53,270	7.53	127	6	3.3	PSMD12	Proteasome, nucleus, cytoplasm
GTPase activation								
Human rab GDI	285975	51,088	5.94	347	15	3.3	RABGDIB	Cytoplasm
Chromatin regulator								
Protein arginine	20070220	73,322	5.88	43	1	3.2	PRMT5	Cytoplasm, nucleus
N-methyltransferase 5								
Glycan metabolism								
Protein kinase C substrate 80K-H isoform 2	48255891	60,110	4.34	51	8	2.6	PRKCSH	Endoplasmic reticulum
Protein disulfide isomerase								
Prolyl 4-hydroxylase, β polypeptide	20070125	57,480	4.76	586	24	2.6	P4HB	Endoplasmic reticulum
Protease inhibitor								
Serine proteinase inhibitor	62898301	42,857	5.90	167	9	2.3	SERPIN	Secreted

**Table II:** Correlation between L-plastin expression and the clinicopathological features of cholangiocarcinoma patients.

	L-plastin expression		
		
Characteristics	Negative	Positive	P-value
Gender			
Male	8	5	1.00
Female	7	4	
Tumor differentiation			
Well	5	3	1.00
Moderate and poor	10	6	
Lymph node metastasis			
No	5	4	0.65
Yes	10	5	
Distant metastasis			
No	11	6	1.00
Yes	4	3	
